# A longitudinal cohort based association study between uric acid level and metabolic syndrome in Chinese Han urban male population

**DOI:** 10.1186/1471-2458-12-419

**Published:** 2012-06-08

**Authors:** Qian Zhang, Chengqi Zhang, Xinhong Song, Haiyan Lin, Dongzhi Zhang, Wenjia Meng, Yongyuan Zhang, Zhenxin Zhu, Fang Tang, Longjian Liu, Xiaowei Yang, Fuzhong Xue

**Affiliations:** 1 Department of Epidemiology and Health Statistics, School of Public Health, Shandong University, PO Box 100, Jinan, 250012, China; 2 Health Management Center, Shandong Provincial QianFoShan Hospital, Jinan, 250014, China; 3 Center for Health Management, Provincial Hospital affiliated to Shandong University, Jinan, China; 4 Department of Epidemiology and Biostatistics, Drexel University School of Public Health, Philadelphia, PA, USA; 5 Department of Public Health Sciences, School of Medicine, University of California, Davis, Davis, CA, 95616, USA

## Abstract

**Background:**

It has been recently demonstrated that serum uric acid (UA) is associated with metabolic syndrome (MetS) or its related clinical indications based on cross-sectional or prospective cohort studies. Nonetheless, due to the fact that UA level constantly fluctuates from time to time even for the person, using a single measure of UA level at baseline of those studies may not be sufficient for estimating the UA-Mets association.

**Methods:**

To further estimate this time-dependent association, we fitted a generalized estimating equation (GEE) regression model with data from a large-scale 6-year longitudinal study, which included 2222 participants aged > =25 years with an average of 3.5 repeated measures of UA per person in the Health Management Center of Shandong Provincial Hospital, Shandong, China.

**Results:**

After adjusting for other potential confounding factors (i.e., total cholesterol, low-density lipoprotein), it was verified that time-dependent UA level was an independent risk factor for MetS (OR = 1.6920, *p* < 0.0001). It was found that UA level was positively associated with obesity, hypertension, and dyslipidemia, but was inversely associated with hyperglycemia.

**Conclusions:**

Serum UA level may serve as an important risk factor of MetS. Additionally, our study suggested that UA level be an independent risk factor to obesity, hypertension and dyslipidemia, but a protective factor to hyperglycemia. These findings are concordant with results from other studies on Asian populations, and jointly provide a basis to further develop a risk assessment model for predicting MetS using UA levels and other factors in China.

## **Background**

Metabolic syndrome (MetS) refers to a combination of medical disorders ,including obesity, dyslipidemia, hypertension and insulin resistance[1]. When occurring together, they increase the risk of developing cardiovascular disease (CVD) and diabetes [2]. Recent studies indicate a wider range of biomarkers of MetS, e.g., alanine aminotransferase, white blood cell count, gamma-glutamyltranspeptidase, and serum uric acid (UA) [3,4]. Based on cross-sectional studies, both positive and negative association between UA levels and MetS has been reported [5-7]. To overcome the inherent limitation of cross-sectional design, a prospective cohort study was conducted recently, which suggested that higher serum UA level would strongly increase the risk of MetS incidence [8]. Nonetheless, such a cohort study with only baseline UA used as predictor is still limited due to the fact that UA level would vary from time to time during the lifespan of most individuals [8]. Therefore, to fully understand the relationship between UA and MetS, and further assess the specific association between UA and each medical disorder in defining MetS, longitudinal studies are expected especially those with large sample sizes and repeated measures of UA. A longitudinal study is usually a cohort study that involves repeated observations of the same set of variables over a period of time. Unlike cross-sectional studies or cohort studies with only baseline measures, a longitudinal study not only tracks each participant’s outcome (e.g., MetS) but also repeatedly measures risk factors that would change with time (e.g., UA). Therefore, longitudinal studies allow one to make observations more accurately and model time-dependent relationships. They are more becoming popular in epidemiology and biomedical research. In this paper, we study the UA-MetS associations using data from a longitudinal study of male residents who visited the Health Management Center of Shandong Provincial Hospital (HMCSPH) for routine physical examinations.

## **Methods**

### **Study population**

In our longitudinal study, participants include 2222 male residents of Shandong Province who visited HMCSPH (an affiliate of organization of Shandong University) at least three times for annual physical examinations between 2005 and 2010. During the 6 year follow-up, each participant on average had 3.63 ± 0.016 measures including serum UA levels. For this study, we only included participants who had not been diagnosed as having MetS at baseline. They were all Chinese Han with baseline age between 25 and 91 years, all belong to middle and upper socioeconomic classes. This study was approved by the Ethics Committee of School of Public Health, Shandong University, and all participants gave informed written consent.

### **Measurements**

Blood samples were collected from participants after an overnight fast of at least 12 hours. Height and weight were measured on participants wearing light clothing without shoes, and body mass index (BMI) was calculated as weight (kg) divided by squared height (m). Blood pressure was measured on right upper arm with participants in sitting position after 5 minutes rest. Peripheral blood samples was obtained for measuring the following parameters: glucose, total cholesterol (CHOL), low-density lipoprotein (LDL), high-density lipoprotein (HDL), triglycerides, uric acid (UA), gamma-glutamyltranspeptidase (GGT), serum albumin (ALB), serum globulins (GLO), blood urea nitrogen (BUN), serum creatinine (CREA), hemoglobin (Hb), hematokrit (HCT), mean corpuscular volume(MCV), mean corpuscular hemoglobin (MCH), Red blood cell distribution width (RDW), white blood count (WBC), platelet distribution width (PDW), mean platelet volume (MPV), and thrombocytocrit (PCT). Data on smoking habits, alcohol intake, diet habits, sleeping quality, exercise frequency, and other variables were obtained using standardized questionnaire.

### **Definition of metS**

Considering the physiological characteristics of our target population, the diagnostic criteria recommended by Diabetes Branch of the Chinese Medical Association (CDS) [9] was used in defining MetS in this study. An participant was claimed as having MetS if he or she had three or more of the following four medical conditions: (1) overweight or obesity, i.e., BMI ≥25.0 Kg/M [2]; (2) hypertension, i.e., systolic blood pressure (SBC) ≥140 mmHg, or diastolic blood pressure (DBP) ≥90 mmHg, or previously diagnosed as hypertension; (3) dyslipidemia, i.e., fasting triglycerides (TG) ≥1.7 mmol/L(110 mg/dl), or fasting high-density (HDL) <0.9 mmol/L(35 mg/dl); (4) hyperglycemia was defined as fasting blood-glucose (FPG) ≥6.1 mmol/L(110 mg/dl), or 2 h Postmeal Glucose (PG) ≥7.8 mmol/L(140 mg/dl),or previously diagnosed as hyperglycemia.

### **Missing data imputation**

In our longitudinal study, there were some variables (see Table 1) with missing values due to early withdrawal of the participants or missing of certain physical examinations. To handle missing values, the strategy of multiple imputation (MI) was adopted where imputations were made using the Markov Chain Monte Carlo (MCMC) algorithm implemented in the MI Procedure of SAS 9.1.3 [10]. Then, each imputed data set was analysed using GEE regression models. Finally the multiple set of estimators for parameters of interest from the GEE analyses were combined to make final inferences. Considering that the variables in our analysis were continuously distributed with arbitrary missingness patterns, a multivariate normal distribution model was assumed in the MI Procedure. As seen in Table 1, most of the imputed variables had less than 10 % of missingness.

**Table 1:** Definition of UA levels and potential confounding factors and missingess rate

**variables**	**Assignments**							**Missingess Rate**
UA	qualified by their quartiles of P_25_, P_50_ and P_75_ every year; **Q1**: the UA level ≤ P_25_, **Q2**: P_25_ < the UA level < P_50_, **Q3**: P_50_ < the UA level < P_75_, **Q4**: the UA level ≥ P_75_							0
		2005	2006	2007	2008	2009	2010	
	P_25_	297	323	302	306	307	323	
	P_50_	353	372	345	349	353	368	
	P_75_	406	419	392	397	401	416	
GGT	gamma-glutamyltranspeptidase , U/L							1 %
ALB	serum albumin, g/L							1 %
GLO	serum globulins, g/L							1 %
BUN	blood urea nitrogen, mg/L							1 %
CREA	serum creatinine, mg/dl							1 %
CHOL	Total cholesterol, mg/dl							1 %
Hb	Hemoglobin, g/L							1 %
HCT	Hematokrit, %							1 %
MCV	mean corpuscular volume, fL							1 %
MCH	mean corpuscular hemoglobin, pg							1 %
RDW	Red blood cell distribution width, %							1 %
WBC	white blood count, 10^9^g/L							1 %
PDW	Platelet distribution width							1 %
MPV	mean platelet volume, fL							1 %
PCT	Thrombocytocrit, %							1 %
diet	0: Vegetarian, 1: meat-based, 2: normal, 3: sea food							17 %
drinking	0: never, 1: seldom, 2: often, wine, 3: often beer, 4: often, Chinese spirits, 5: often, mixed all kinds							6 %
smoking	0: never, 1: seldom, 2: quit, 3: 1-4/d, 4: 5-15/d, 5: >15/d							6 %
quality of sleep	0: excellent, 1: well, 2: fair, 3: poor, 4: very poor							6 %
exercise	0: never, 1: seldom, 2: often or everyday							6 %

### **UA Levels and potential confounding factors**

Before fitting a GEE model, the original continuous serum UA measure was categorized into 4 levels using the 3 quartiles (P_25_, P_50_ and P_75_) as cut-off values. As seen in Table 1, potential confounding factors were also considered during the GEE analysis.

### **Statistical analysis**

Descriptive analyses were first conducted for the distributions of UA levels and potential covariates collected at the baseline survey. Then, each variable of interest was compared, using student’s t-test, between subjects with and subjects without MetS at each follow-up interval (i.e., each year baseline). To study the association between UA levels and MetS, we first fitted simple GEE models, each with a single predictor (i.e., the UA variable or any one of the confounding factors). We finally fitted a multiple GEE regression model. A covariate was added to the final multiple regression model only if it was found significant (p < 0.05) in the single-predictor model. Note that the GEE modelling strategy is capable of describing time-dependent relationship between UA levels and the MetS status (yes or no), after adjusting potential confounders. All these variables are repeatedly measured during the 6-year follow-up. Age at baseline was also added to the multiple GEE analysis procedure to adjust for the age effect. In each GEE model, ‘Logit’ was chosen as the link function of GEE, because either MetS status is a binary dependent variable. Following the same procedure, we studied the association between UA levels and each one of the four medical disorders in defining MetS (i.e., overweight/obesity, hypertension, dyslipidemia, and hyperglycemia), which were all defined as binary variables. All statistical analysis was performed by SAS 9.1.3.

## **Results**

Additional file 1: Table S1 in Supplement Materials shows the distribution of UA levels and other potential confounding factors at the baseline survey, and their distributions between subjects with and subjects without MetS at each follow-up interval (i.e., each year after baseline). The table shows that the UA level and other potential confounding factors in the MetS group were generally higher than their counterparts in the non-MetS group at each follow-up intervals (P < 0.05), although some factors were not statistically significant at some follow-up intervals. The numbers of participants at each follow-up interval during the study period (Jan 2005 - Dec 2010) are also shown in the Supplemental Materials; see Additional file 1: Table S2.

Table 2 shows the results of single-predictor GEE models for the UA levels and other potential confounding factors. It is seen that the UA level was strongly associated with MetS (OR = 1.2952; 95 % CI = 1.1864-1.4141; p < .0001). Age, GGT, ALB, GLO, CHOL, Hb, MCV and MCH and WBC were all significant predictors or confounders at the α = 0.05 level. Other factors including BUN, CREA, HCT, RDW, PDW, MPV, PCT, iet, drinking, smoking, quality of sleep and exercise are not significant at the α = 0.05 level.

**Table 2:** Single-predictor GEE Models

	**Estimate**	**Standard Error**	**Z**	**Pr > |Z|**	**RR**	**lower 95% Confidence Limits**	**upper 95% Confidence Limits**
**Quartiles of UA**	0.2587	0.0448	5.78	<.0001	1.2952	1.1864	1.4141
**age**	0.0243	0.0031	7.94	<.0001	1.0246	1.0185	1.0308
**GGT**	0.0095	0.0012	8.15	<.0001	1.0095	1.0072	1.0118
**ALB**	0.037	0.0097	3.8	0.0001	1.0377	1.0181	1.0576
**GLO**	−0.0574	0.0172	−3.33	0.0009	0.9442	0.9128	0.9766
BUN	−0.0083	0.0431	−0.19	0.8474	0.9917	0.9114	1.0792
CREA	−0.0004	0.0053	−0.08	0.933	0.9996	0.9894	1.0099
**CHOL**	0.3292	0.0491	6.7	<.0001	1.3899	1.2623	1.5304
**Hb**	0.0242	0.0052	4.67	<.0001	1.0245	1.0142	1.0350
HCT	0.0119	0.0173	0.69	0.4913	1.0120	0.9782	1.0470
**MCV**	−0.0296	0.0112	−2.64	0.0083	0.9708	0.9496	0.9924
**MCH**	0.0905	0.0286	3.17	0.0015	1.0947	1.0351	1.1579
RDW	−0.0613	0.0674	−0.91	0.3632	0.9405	0.8242	1.0734
**WBC**	0.1391	0.0276	5.05	<.0001	1.1492	1.0888	1.2130
PDW	0.0045	0.0299	0.15	0.8795	1.0045	0.9474	1.0651
MPV	−0.0034	0.0623	−0.05	0.9563	0.9966	0.8821	1.1259
PCT	−1.4813	1.0857	−1.36	0.1725	0.2273	0.0271	1.9092
diet	−0.0248	0.0535	−0.46	0.6433	0.9755	0.8784	1.0834
drinking	0.0172	0.029	0.59	0.5539	1.0173	0.9611	1.0768
smoking	0.0044	0.0241	0.18	0.8536	1.0044	0.9582	1.0530
quality of sleep	−0.0242	0.062	−0.39	0.696	0.9761	0.8644	1.1021
exercise	0.015	0.103	0.15	0.8841	1.0151	0.8295	1.2423

Table 3 illustrates the association between UA levels and MetS status after adjusting other potential confounding factors using the final multiple GEE model. It reveals that, compared with the lowest level of UA (Q1), the highest level of UA (Q4) was strongly associated with MetS status with a positive relationship (OR = 1.6920; 95 % CI = 1.3390-2.1381; *p* < 0.0001). Although not significant, the ORs for Q2 and Q3 were all larger than 1.0, and an increasing trend was seen in OR from Q2 to Q4.

**Table 3:** The results of multiple GEE analysis for UA levels and MetS after adjusting other potential confounding factors

	**Estimate**	**Error**	**Z**	**Pr > |Z|**	**RR**	**lower 95 % Confidence Limits**	**upper 95 % Confidence Limits**
Intercept	−5.3378	1.4182	−3.76	0.0002			
**Q4**	**0.5259**	**0.1194**	**4.41**	**<.0001**	**1.6920**	**1.3390**	**2.1381**
Q3	0.1856	0.1244	1.49	0.1357	1.2039	0.9435	1.5363
Q2	0.0709	0.1274	0.56	0.5778	1.0735	0.8363	1.3780
Q1	0	0	ref	ref	ref	ref	ref
**time**	**0.3072**	**0.026**	**11.84**	**<.0001**	**1.3596**	**1.2923**	**1.4306**
**baseage**	**0.0308**	**0.0034**	**9.16**	**<.0001**	**1.0313**	**1.0245**	**1.0381**
**GGT**	**0.0091**	**0.0011**	**7.96**	**<.0001**	**1.0091**	**1.0069**	**1.0115**
ALB	−0.018	0.018	−1	0.3197	0.9822	0.9481	1.0176
**GLO**	**0.0228**	**0.0103**	**2.21**	**0.0268**	**1.0231**	**1.0026**	**1.0439**
**CHOL**	**0.1285**	**0.0468**	**2.74**	**0.0061**	**1.1371**	**1.0374**	**1.2463**
**Hb**	**0.012**	**0.0046**	**2.63**	**0.0086**	**1.0121**	**1.0031**	**1.0212**
**MCV**	**−0.1321**	**0.019**	**−6.96**	**<.0001**	**0.8763**	**0.8443**	**0.9095**
**MCH**	**0.2862**	**0.0462**	**6.19**	**<.0001**	**1.3314**	**1.2160**	**1.4575**
**WBC**	**0.1531**	**0.0242**	**6.33**	**<.0001**	**1.1654**	**1.1115**	**1.2220**

**Table 4:** The results of multiple GEE analysis for UA levels and Obesity after adjusting other potential confounding factors

**Quartiles**	**Estimate**	**Error**	**Z**	**Pr > |Z|**	**RR**	**lower 95 % Confidence Limits**	**upper 95 % Confidence Limits**
Intercept	−0.3359	0.8418	−0.4	0.6899			
**Q4**	**0.9224**	**0.0695**	**13.28**	**<.0001**	**2.5153**	**2.1953**	**2.8823**
**Q3**	**0.7133**	**0.0653**	**10.92**	**<.0001**	**2.0407**	**1.7955**	**2.3194**
**Q2**	**0.4271**	**0.0634**	**6.73**	**<.0001**	**1.5328**	**1.3536**	**1.7357**
Q1	0	0	ref	ref	ref	ref	ref
**hypertension**	**0.1446**	**0.0581**	**2.49**	**0.0128**	**1.1556**	**1.0312**	**1.2950**
**dyslipidemia**	**0.2913**	**0.0484**	**6.02**	**<.0001**	**1.3382**	**1.2170**	**1.4712**
hyperglycemia	−0.0503	0.0778	−0.65	0.5176	0.9509	0.8164	1.1075
time	−0.0117	0.0191	−0.61	0.5417	0.9884	0.9520	1.0261
**baseage**	**0.0055**	**0.0023**	**2.42**	**0.0155**	**1.0055**	**1.0010**	**1.0101**
**GGT**	**0.0061**	**0.0013**	**4.86**	**<.0001**	**1.0061**	**1.0036**	**1.0085**
**ALB**	**−0.044**	**0.0096**	**−4.56**	**<.0001**	**0.9570**	**0.9390**	**0.9752**
GLO	−0.0075	0.0063	−1.2	0.2319	0.9925	0.9805	1.0048
CHOL	0.0115	0.0269	0.43	0.6702	1.0116	0.9595	1.0663
**Hb**	**0.0188**	**0.0028**	**6.81**	**<.0001**	**1.0190**	**1.0135**	**1.0245**
MCV	−0.0147	0.0109	−1.34	0.179	0.9854	0.9646	1.0067
MCH	−0.0155	0.0264	−0.59	0.5574	0.9846	0.9349	1.0370
**WBC**	**0.0597**	**0.0162**	**3.68**	**0.0002**	**1.0615**	**1.0283**	**1.0959**

**Table 5:** The results of multiple GEE analysis for UA levels and hypertension after adjusting other potential confounding factors

	**Estimate**	**Error**	**Z**	**Pr > |Z|**	**RR**	**lower 95 % Confidence Limits**	**upper 95 % Confidence Limits**
Intercept	−5.9514	1.0022	−5.94	<.0001			
**Q4**	**0.3953**	**0.0822**	**4.81**	**<.0001**	**1.4848**	**1.2639**	**1.7444**
**Q3**	**0.1751**	**0.0804**	**2.18**	**0.0294**	**1.1914**	**1.0178**	**1.3947**
Q2	−0.0189	0.0808	−0.23	0.8146	0.9813	0.8375	1.1496
Q1	0	0	ref	ref	ref	ref	ref
**hyperglycemia**	**0.345**	**0.0843**	**4.09**	**<.0001**	**1.4120**	**1.1969**	**1.6658**
**dyslipidemia**	**−0.3991**	**0.0596**	**−6.69**	**<.0001**	**0.6709**	**0.5969**	**0.7541**
**obesity**	**0.1882**	**0.0584**	**3.22**	**0.0013**	**1.2071**	**1.0766**	**1.3534**
**time**	**0.101**	**0.0218**	**4.64**	**<.0001**	**1.1063**	**1.0600**	**1.1544**
**baseage**	**0.0686**	**0.0027**	**25.55**	**<.0001**	**1.0710**	**1.0655**	**1.0767**
**GGT**	**0.0077**	**0.001**	**7.62**	**<.0001**	**1.0077**	**1.0057**	**1.0097**
ALB	0.0028	0.0119	0.23	0.8149	1.0028	0.9797	1.0263
**GLO**	**0.0155**	**0.0071**	**2.19**	**0.0285**	**1.0156**	**1.0016**	**1.0298**
CHOL	0.0216	0.0314	0.69	0.4923	1.0218	0.9608	1.0867
**Hb**	**0.0135**	**0.0031**	**4.31**	**<.0001**	**1.0136**	**1.0074**	**1.0198**
**MCV**	**−0.0276**	**0.0131**	**−2.1**	**0.0357**	**0.9728**	**0.9481**	**0.9982**
MCH	0.0114	0.032	0.36	0.722	1.0115	0.9500	1.0768
**WBC**	**0.0538**	**0.0186**	**2.8900**	**0.0039**	**1.0553**	**1.0175**	**1.0945**

The results on association of UA levels with each single MetS component, are shown in Table 4, 5, 6 and 7 of the Supplemental Materials, respectively for overweight/obesity, hypertension dyslipidemia and hyperglycemia. Table 4 shows that UA was a strong independent risk factor to overweight/obesity; and an obvious increasing trend in OR is seen from Q2 to Q4 after adjusting the other three components of MetS and potential confounding factors. Table 6 and 4 jointly depict similar patterns for the association between UA levels and hypertension or dyslipidemia, except that the OR is not significant at the Q2 level. In contrast, UA is identified as a protective factor with the range of ORs ranged from 0.58-0.74 after adjusting the other three components and the same set of potential confounding factors; see Table 7.

**Table 6:** The results of multiple GEE analysis for UA levels and dyslipidemia after adjusting other potential confounding factors

	**Estimate**	**Error**	**Z**	**Pr > |Z|**	**RR**	**lower 95 % Confidence Limits**	**upper 95 % Confidence Limits**
Intercept	−0.4838	0.8527	−0.57	0.5704			
**Q4**	**0.4752**	**0.0713**	**6.66**	**<.0001**	**1.6083**	**1.3985**	**1.8498**
**Q3**	**0.1815**	**0.067**	**2.71**	**0.0068**	**1.1990**	**1.0514**	**1.3672**
Q2	0.0526	0.0659	0.8	0.4247	1.0540	0.9264	1.1993
Q1	0	0	ref	ref	ref	ref	ref
**hyperglycemia**	**0.1725**	**0.0795**	**2.17**	**0.03**	**1.1883**	**1.0168**	**1.3886**
**hypertension**	**−0.4309**	**0.0603**	**−7.15**	**<.0001**	**0.6499**	**0.5775**	**0.7315**
**obesity**	**0.2916**	**0.0485**	**6.02**	**<.0001**	**1.3386**	**1.2173**	**1.4720**
**time**	**−0.23**	**0.0196**	**−11.75**	**<.0001**	**0.7945**	**0.7646**	**0.8256**
**baseage**	**−0.009**	**0.0023**	**−3.86**	**0.0001**	**0.9910**	**0.9865**	**0.9956**
**GGT**	**0.0106**	**0.0013**	**8.39**	**<.0001**	**1.0107**	**1.0081**	**1.0131**
ALB	0.0005	0.01	0.05	0.9605	1.0005	0.9810	1.0204
**GLO**	0.0101	0.0063	1.59	0.1109	1.0102	0.9977	1.0229
**CHOL**	−0.0465	0.027	−1.72	0.085	0.9546	0.9054	1.0064
**Hb**	**0.0108**	**0.0027**	**3.96**	**<.0001**	**1.0109**	**1.0055**	**1.0163**
**MCV**	**−0.097**	**0.0114**	**−8.51**	**<.0001**	**0.9076**	**0.8875**	**0.9281**
**MCH**	**0.2093**	**0.0278**	**7.53**	**<.0001**	**1.2328**	**1.1674**	**1.3017**
**WBC**	**0.163**	**0.0166**	**9.8**	**<.0001**	**1.1770**	**1.1393**	**1.2160**

## **Discussions**

The relationship among UA, MetS and cardiovascular diseases has received much attention in recent years. The findings from a nationwide representative sample of US adults indicate that the prevalence of the MetS increases substantially with increasing levels of UA [11]. UA as an independent risk predictor for MetS also was found in Korean male workers aged 30–39 years [12], in Thai adults receiving annual physical examinations [13], in Japanese adolescents [14], in an urban population in Turkey [15], and in many other populations. Particularly, the debate on whether UA should be viewed even as an additional component of MetS received intensive discussions [16]; and the relationship between UA and each MetS component was also controversial. In this paper, based on a large longitudinal cohort study over 6 years, we confirmed that UA level is positively and significantly associated with risk of MetS among healthy male adults in Han Chinese of Shandong province, after adjusting other potential confounding factors using GEE regression analyses. This provides us a strong piece of evidence for developing risk assessment model for early screening of MetS.

As an essential component of MetS, obesity was regarded as a main contributor of the increasing prevalence of MetS, because obesity was proved as a risk factor of hypertension, dyslipidemia and hyperuricemia [17]. The positive gradient of obesity with increasing UA level has been found among several recent studies [18,19]. In this study, we found consistent results: in Chinese Han urban male population from middle to upper socioeconomic classes, UA levels of Q2, Q3, Q4 correspond to relative risks of 1.53, 2.04, and 2.51, respectively, in getting obesity (with Q1 as reference level; see Table 7. Additionally we found that the effect of UA on obesity become much larger, compared to MetS; see Table 3. In pathophysiology, it was explained in three ways for the fact that obesity causes higher level of UA in blood: (1) obesity directly interferes with urate synthesis and excretion [20]; (2) obesity causes renal damage via glomerulus dysfunction [21]; (3) obesity leads to dysfunction of the renin-angiotensin system, which would eventually results in fractional clearance of UA [22].

**Table 7:** The results of multiple GEE analysis for UA levels and hyperglycemia after adjusting other potential confounding factors

**Quartiles**	**Estimate**	**Error**	**Z**	**Pr > |Z|**	**RR**	**lower 95 % Confidence Limits**	**upper 95 % Confidence Limits**
Intercept	−3.9156	1.2888	−3.04	0.0024			
**Q4**	**−0.3752**	**0.1069**	**−3.51**	**0.0004**	**0.6872**	**0.5573**	**0.8473**
**Q3**	**−0.5427**	**0.1084**	**−5.01**	**<.0001**	**0.5812**	**0.4700**	**0.7188**
**Q2**	**−0.3063**	**0.1026**	**−2.98**	**0.0028**	**0.7362**	**0.6021**	**0.9002**
Q1	0	0	ref	ref	ref	ref	ref
**hypertension**	**0.3269**	**0.0875**	**3.74**	**0.0002**	**1.3867**	**1.1681**	**1.6461**
**dyslipidemia**	**0.1672**	**0.0799**	**2.09**	**0.0363**	**1.1820**	**1.0108**	**1.3822**
obesity	0.007	0.0774	0.09	0.9277	1.0070	0.8652	1.1721
**time**	**0.2352**	**0.0279**	**8.43**	**<.0001**	**1.2652**	**1.1978**	**1.3363**
**baseage**	**0.0327**	**0.0033**	**10.03**	**<.0001**	**1.0332**	**1.0266**	**1.0399**
**GGT**	**0.0056**	**0.001**	**5.59**	**<.0001**	**1.0056**	**1.0036**	**1.0076**
ALB	0.0218	0.016	1.36	0.1735	1.0220	0.9904	1.0545
GLO	−0.0014	0.0093	−0.15	0.8834	0.9986	0.9805	1.0170
**CHOL**	**0.2507**	**0.0405**	**6.19**	**<.0001**	**1.2849**	**1.1868**	**1.3911**
**Hb**	**−0.0101**	**0.0041**	**−2.47**	**0.0136**	**0.9900**	**0.9820**	**0.9979**
**MCV**	**−0.0935**	**0.0186**	**−5.04**	**<.0001**	**0.9107**	**0.8782**	**0.9445**
**MCH**	**0.2233**	**0.0436**	**5.12**	**<.0001**	**1.2502**	**1.1476**	**1.3618**
**WBC**	**0.0753**	**0.0236**	**3.1900**	**0.0014**	**1.0782**	**1.0294**	**1.1293**

The association between hyperglycemia and UA, however, still remained controversy. Most researches suggested that hyperglycemia and UA should be the pathophysiological underpinning of MetS a long time ago [23], and a number of prospective studies have provided evidence that individuals with higher UA are at a higher risk of diabetes [9,24,25]. Nonetheless, some studies claimed that UA is negatively associated with diabetes in Asian men [7,12], and an inverse association between UA and fasting plasma glucose was also observed in adult residents of Taiwan [6]. In our study, also in Asian male population, Q2-Q4 of UA was found to have lower risk of hyperglycemia with adjusted OR from 0.58-0.74; see Table 5. This protective effect can be explained in two ways: (1) UA-hyperglycemia association might be gender-dependent among Asian populations (i.e., UA is a risk factor in females, but a protective factor in males); (2) the relationship might be nonlinear and hyperglycemia could impair tubular reabsorption of UA [6,26].

UA was first demonstrated to be associated with hypertension by Mohamed in 1870 s [27], and in 2008, based on the large population in the Normative Aging Study. The baseline UA was reported as a durable marker of risk of hypertension [28]. In our longitudinal cohort study with 6 years of follow up, the GEE analyses confirmed that UA is an independent risk factor of hypertension in Chinese Han urban males; see Table 6. As found by Watanabe et al. [29], once elevated UA level causes sufficient renal injury, human body would develop salt-sensitive hypertension. Elevated UA also associates with increased amount of free radicals [30] and an oxidative stress, which may further abolish endothelium-dependent vasodilatation [31].

Finally, it has been well received that elevated UA level would increase the risk of dyslipidemia [12,30]. Our longitudinal study also suggests a positive association between UA dyslipidemia; see Table 4. Although the role of UA in the metabolism of triglycerides and other lipids still remains unclear, it was believed that UA might be involved in either the overproduction or the reduction of clearance of lipids [32].

## Conclusions

Association between UA and cardiovascular diseases has been reported frequently recent years. Many research confirmed that UA was a strong risk factor for cardiovascular diseases, especially MetS [33-35]. Our longitudinal study further verified that UA is an independent risk factor of MetS, higher level of UA leading to higher risk of MetS. All the analyses were based on GEE regression models with data from the longitudinal cohort study with Han Chinese urban male participants who were from middle to upper socioeconomic classes. Additionally, our study suggested that UA level be an independent risk factor to obesity, hypertension and dyslipidemia, but a protective factor to hyperglycemia. These findings are concordant with results from other studies on Asian populations, and jointly provide a basis to further develop a risk assessment model for predicting MetS using UA levels and other factors in China.

There were several limitations in the present study. Due to the fact that participants of the study who came to the hospitals mainly for physical examinations, they were mandatorily asked to report histories on medication for treating MetS or related medical disorders (e.g., hypertension). Thus we did not have data on medication in our GEE regression analyses in studying the UA–MetS association. Also considering that our study contains only local males from relatively rich and educated families in Shandong province, and the fact that our analyses only covered a follow-up period of 6 years and no genetic information was included, it is expected a large scale longitudinal study is expected for a better and fully understanding of the target relationships.

## Competing interests

There is no conflict of interest for any of the authors. All authors had access to the data and were involved in drafting the article and revising it critically for important intellectual content.

## Authors' contributions

In our work, FX and CZ designed the study and directed its implementation, including quality assurance and control. XS, FT, HL and DZ did the clinical exam and collected the data. WM, YZ and ZZ helped analyzing the data. LL provided scientific comments and advice. XY worked on writing of the paper. QZ participated much of the above work and led the writing of the paper. All authors read and approved the final manuscript

## Pre-publication history

The pre-publication history for this paper can be accessed here:

http://www.biomedcentral.com/1471-2458/12/419/prepub

## Supplementary Material

Additional file 1 **Table S1.** The distribution of UA levels and other potential confounding factors. Table S2 Numbers Participants at Each Year of the Study Click here for file
