# Supplementary material for: A longitudinal cohort based association study between uric acid level and metabolic syndrome in Chinese Han urban male population
Source: BMC Public Health. 2012 Jun 8;12:419. doi: 10.1186/1471-2458-12-419 (PMC3416673; doi:10.1186/1471-2458-12-419)
Supplement: Additional file 1 — Table S1. The distribution of UA levels and other potential confounding factors. Table S2 Numbers Participants at Each Year of the Study [file 1471-2458-12-419-S1.doc]

**S-Table1** the distribution of UA levels and other potential confounding factors

|  |  | **baseline** | **1 year after baseline** | | **2 year after baseline** | | **3 year after baseline** | | **4 year after baseline** | | **5 year after baseline** | |
| --- | --- | --- | --- | --- | --- | --- | --- | --- | --- | --- | --- | --- |
| **Variable** | **MS** | **Mean±Std** | **N** | **Mean±Std** | **N** | **Mean±Std** | **N** | **Mean±Std** | **N** | **Mean±Std** | **N** | **Mean±Std** |
| **UA** | 0 | 356.03 ±72.08 | 1632 | 353.28 ±69.82^*^ | 1831 | 356.54 ±70.37^*^ | 1041 | 365.75 ±73.54^*^ | 494 | 363.54 ±75.27 | 132 | 365.72 ±66.26 |
|  | 1 |  | 206 | 368.84 ±77.68^*^ | 254 | 382.88 ±81.05^*^ | 164 | 390.74 ±82.77^*^ | 73 | 381.19 ±75.25 | 16 | 378.88 ±96.04 |
| **age** | 0 | 46.51 ±12.29 | 1632 | 47.11 ±12.46^*^ | 1831 | 48.28 ±12.39^*^ | 1041 | 50.01 ±13.02^*^ | 494 | 53.74 ±13.48^*^ | 132 | 54.05 ±13.93 |
|  | 1 |  | 206 | 51.99±11.97^*^ | 254 | 52.50 ±11.29^*^ | 164 | 52.22 ±11.49^*^ | 73 | 58.68 ±13.58^*^ | 16 | 54.13 ±12.39 |
| **GGT** | 0 | 32.99±26.94 | 1632 | 32.81±27.18^*^ | 1831 | 31.88 ±24.73^*^ | 1041 | 32.04 ±24.85^*^ | 494 | 32.76 ±28.76^*^ | 132 | 29.77 ±17.03 |
|  | 1 |  | 206 | 43.13±38.94^*^ | 254 | 44.44 ±55.36^*^ | 164 | 46.45 ±33.77^*^ | 73 | 45.15 ±40.29^*^ | 16 | 37.38 ±19.23 |
| **ALB** | 0 | 46.55 ±2.55 | 1632 | 45.79±2.79 | 1831 | 45.55 ±2.67 | 1041 | 45.22 ±2.49 | 494 | 44.92 ±2.65 | 132 | 45.79 ±2.36 |
|  | 1 |  | 206 | 45.65±2.71 | 254 | 45.32 ±2.52 | 164 | 45.35 ±2.64 | 73 | 44.98 ±2.58 | 16 | 45.91 ±2.45 |
| **GLO** | 0 | 27.16 ±3.73 | 1632 | 27.69±4.14^*^ | 1831 | 28.64 ±3.84^*^ | 1041 | 29.31 ±3.87^*^ | 494 | 29.75 ±3.84 | 132 | 30.69 ±3.24 |
|  | 1 |  | 206 | 28.29±4.19^*^ | 254 | 29.97 ±3.95^*^ | 164 | 30.27 ±5.11^*^ | 73 | 30.30 ±4.05 | 16 | 30.41 ±3.25 |
| **BUN** | 0 | 5.33 ±1.15 | 1632 | 5.24 ±1.13 | 1831 | 5.26 ±1.14 | 1041 | 5.38 ±1.21 | 494 | 5.34 ±1.25 | 132 | 5.63 ±1.68 |
|  | 1 |  | 206 | 5.29 ±1.33 | 254 | 5.31 ±1.17 | 164 | 5.47 ±1.34 | 73 | 5.52 ±1.07 | 16 | 5.32 ±1.12 |
| **CREA** | 0 | 88.75±9.94 | 1632 | 88.52 ±9.53 | 1831 | 88.17 ±9.95 | 1041 | 89.07 ±10.66 | 494 | 88.77 ±12.03 | 132 | 89.29 ±16.41 |
|  | 1 |  | 206 | 88.93 ±11.74 | 254 | 88.52 ±11.19 | 164 | 89.03 ±12.32 | 73 | 90.43 ±13.03 | 16 | 83.79 ±7.89 |
| **CHOL** | 0 | 5.10 ±0.90 | 1632 | 5.11 ±0.92^*^ | 1831 | 5.21 ±0.93^*^ | 1041 | 5.27 ±0.92^*^ | 494 | 5.30 ±0.86 | 132 | 5.55 ±0.81 |
|  | 1 |  | 206 | 5.36 ±1.11 | 254 | 5.48 ±1.19^*^ | 164 | 5.62 ±1.14^*^ | 73 | 5.24 ±1.05 | 16 | 5.39 ±0.85 |
| **Hb** | 0 | 155.33 ±9.95 | 1632 | 155.25 ±9.99 | 1831 | 155.23 ±10.35^*^ | 1041 | 155.35 ±11.08^*^ | 494 | 153.89 ±10.63^*^ | 132 | 154.78 ±11.64 |
|  | 1 |  | 206 | 156.63 ±10.47 | 254 | 157.26 ±9.91^*^ | 164 | 157.35 ±11.20 | 73 | 156.92 ±12.25^*^ | 16 | 160.19 ±6.07 |
| **HCT** | 0 | 45.72 ±2.71 | 1632 | 45.47 ±2.85 | 1831 | 45.18 ±2.86 | 1041 | 45.18 ±3.03 | 494 | 44.52 ±2.89^*^ | 132 | 44.69 ±2.93 |
|  | 1 |  | 206 | 45.67 ±2.91 | 254 | 45.33 ±2.77 | 164 | 45.26 ±3.07 | 73 | 45.20 ±3.23^*^ | 16 | 45.61 ±2.37 |
| **MCV** | 0 | 90.54 ±3.98 | 1632 | 90.48 ±4.00 | 1831 | 90.16 ±4.12 | 1041 | 90.19 ±4.05 | 494 | 90.00 ±4.15 | 132 | 89.82 ±3.83 |
|  | 1 |  | 206 | 90.52 ±3.87 | 254 | 89.60 ±3.73 | 164 | 89.82 ±3.69 | 73 | 90.16 ±3.33 | 16 | 88.41 ±3.21 |
| **MCH** | 0 | 30.76 ±1.56 | 1632 | 30.91 ±1.70 | 1831 | 30.99 ±1.70 | 1041 | 31.01 ±1.67 | 494 | 31.12 ±1.74 | 132 | 31.10 ±1.55 |
|  | 1 |  | 206 | 31.05 ±1.61 | 254 | 31.09 ±1.45 | 164 | 31.23 ±1.45 | 73 | 31.29 ±1.47 | 16 | 31.08 ±1.38 |
| **RDW** | 0 | 12.75 ±0.68 | 1632 | 341.61 ±11.14 | 1831 | 343.65 ±10.61^*^ | 1041 | 343.90 ±10.95^*^ | 494 | 345.73 ±10.35 | 132 | 346.13 ±8.30^*^ |
|  | 1 |  | 206 | 343.02 ±10.53 | 254 | 347.03 ±10.09^*^ | 164 | 347.70 ±10.68^*^ | 73 | 347.08 ±10.37 | 16 | 351.50 ±10.39^*^ |
| **WBC** | 0 | 6.55 ±1.52 | 1632 | 6.47 ±1.49^*^ | 1831 | 6.45 ±1.47^*^ | 1041 | 6.50 ±1.58^*^ | 494 | 6.49 ±1.50^*^ | 132 | 6.38 ±1.58 |
|  | 1 |  | 206 | 6.79 ±1.37^*^ | 254 | 6.92 ±1.55^*^ | 164 | 7.09 ±1.59^*^ | 73 | 7.14 ±1.85^*^ | 16 | 6.72 ±1.28 |
| **PDW** | 0 | 10.39 ±0.82 | 1632 | 12.26 ±1.74 | 1831 | 12.19 ±1.71 | 1041 | 12.11 ±1.67 | 494 | 12.01 ±1.66 | 132 | 12.01 ±1.59 |
|  | 1 |  | 206 | 12.29 ±2.03 | 254 | 12.18 ±1.81 | 164 | 12.29 ±1.75 | 73 | 12.05 ±1.83 | 16 | 12.37 ±1.81 |
| **MPV** | 0 | 0.24 ±0.05 | 1632 | 10.35 ±0.82 | 1831 | 10.36 ±0.81 | 1041 | 10.38 ±0.81 | 494 | 10.33 ±0.81 | 132 | 10.43 ±0.76 |
|  | 1 |  | 206 | 10.33 ±0.86 | 254 | 10.34 ±0.86 | 164 | 10.44 ±0.83 | 73 | 10.32 ±0.91 | 16 | 10.53 ±0.87 |
| **PCT** | 0 | 28.09 ±6.38 | 1632 | 0.237 ±0.047^*^ | 1831 | 0.234 ±0.047^*^ | 1041 | 0.229 ±0.047^*^ | 494 | 0.225 ±0.047^*^ | 132 | 0.226 ±0.049^*^ |
|  | 1 |  | 206 | 0.238 ±0.055 | 254 | 0.233 ±0.046^*^ | 164 | 0.232 ±0.046^*^ | 73 | 0.212 ±0.048^*^ | 16 | 0.222 ±0.043^*^ |

* P<0.05

**S-Table 2 Numbers Participants at Each Year of the Study**

|  | **2005** | **2006** | **2007** | **2008** | **2009** | **2010** |
| --- | --- | --- | --- | --- | --- | --- |
| **No.of MetS** | 0 | 10  (1.72%) | 82  (5.36%) | 120  (5.82%) | 256  (12.98%) | 245  (15.27%) |
| **total** | 315 | 581 | 1531 | 2061 | 1973 | 1604 |
